# An analysis of FDA drug approvals for oncological hematological malignancies in relation to companion diagnostics

**DOI:** 10.3389/fonc.2025.1635491

**Published:** 2025-09-25

**Authors:** Jan Trøst Jørgensen

**Affiliations:** Medical Science, Dx-Rx Institute, Fredensborg, Denmark

**Keywords:** FDA approvals, oncology, hematology, targeted therapy, tissue-agnostic drugs, companion diagnostics

## Introduction

Over the last few decades, our understanding of the pathophysiology of oncological and hematological malignancies has increased considerably. Recognizing the role of oncogenic drivers and the considerable intra- and inter-tumor heterogeneity in human cancers requires a more individualized treatment approach (1, 2). The improved insight into tumor biology has facilitated the development of therapies that target specific molecular components involved in essential cellular processes, thereby preventing proliferation and survival of cancer cells (3). The selection of patients for this type of therapy is often based on the identification of specific molecular characteristics to determine treatment eligibility.

The development of trastuzumab marked the first instance in which a molecular predictive assay was developed alongside a targeted drug and utilized for patient selection. The importance of this drug-diagnostics co-development model has been emphasized by former ASCO president Gabriel Hortobagyi, who stated that if an assay did not exist to identify the patient population likely to respond to therapy, trastuzumab might have been discarded during development because of insufficient activity in an unselected patient population (4). In 1998, trastuzumab, together with its immunohistochemical (IHC) assay HercepTest, was approved by the Food and Drug Administration (FDA) for the treatment of metastatic HER2 positive breast cancer (5). Subsequently, regulatory bodies have termed this type of predictive biomarker assay linked to a specific drug or group of drugs a companion diagnostic (CDx) (6, 7). The FDA defines CDx as an *in vitro* diagnostic assay or imaging tool that provides information that is essential for the safe and effective use of a corresponding therapeutic product (7). Similar definitions have been established by regulatory bodies in Europe, Japan, and other countries (8, 9). In most instances, CDx assays are developed concurrently with drugs or biological products to achieve simultaneous regulatory approval. This is crucial because the CDx assay needs to be available to clinicians simultaneously with the drug, enabling selection of the right patient population for treatment.

Since the monoclonal antibody trastuzumab received approval more than 25 years ago, there has been a consistent increase in the number of drugs and biological agents that are linked to a CDx assays. By early 2025, the FDA had approved more than 78 drug/CDx combinations (10). It is no longer only antibody-based drugs that are guided by a CDx assay. Today, a number of other drug classes, such as kinase inhibitors, antibody-drug conjugates (ADC), and various small-molecule drugs, have a CDx linked to their use (10). The aim of this brief report is to describe the growth in the number of new molecular entities (NME) linked to companion diagnostic (CDx) assays from 1998 to the end of 2024 based on publicly available information from the FDA on drug, biological, and CDx approvals.

## Methods

Various listings and databases were systematically examined to identify the NMEs approved by the FDA from 1998 to 2024, employing the following six steps. 1) For NMEs approved in the period from 2006 to 2024, the data available in the listings on “Oncology (Cancer)/Hematologic Malignancies Approval Notifications” were extracted (11). 2) For the remaining period 1998–2005 the facility of searching for original the New Drug Application (NDA) and Biologics License Application (BLA) approvals by month of the “Drugs@FDA” database was used (10). 3) In addition to these listings and databases the list on “Approved Cellular and Gene Therapy Products” were scoured (12). 4) For all drugs and biologics identified via these listings and databases, their individual full prescribing information was reviewed, and special attention was paid to the subsection “Patient Selection” under paragraph “Dosage and Administration” (10). 5) All drugs and biological agents identified were subsequently verified with respect to their CDx assay using the FDA “List of Cleared or Approved Companion Diagnostic Devices (*In Vitro* and Imaging Tools)” (13). The data extracted from different databases covered only the NME with respect to the initial NDA/BLA. 6) However, when assessing whether a drug had a CDx linked to its use at a later point, information from the supplemental NDA/BLAs was reviewed based on the information available in the “Drugs@FDA” database for the individual NMEs, under section “Approval Date(s), and History, Letters, Labels and Reviews” (10).

The collected information was compiled into an Excel sheet that also contained the exported information from the FDA “List of Cleared or Approved Companion Diagnostic Devices (*In Vitro* and Imaging Tools)” (13). The identified NME were further categorized using the molecular/therapeutic classification available in the full prescribing information for each drug (10). This classification comprises the following eight categories: Kinase Inhibitors, Antibodies, Small-molecule Drugs, Chemotherapeutics, Advanced Therapy Medical Products (ATMP), Antibody-Drug Conjugates (ADC), Radiopharmaceuticals, and Others. Due to the critical role of the CDx assay in relation to tissue agnostic drugs, specific focus is given to the type of drugs.

## Outcome analysis

### Drug approvals

**Figure 1:**
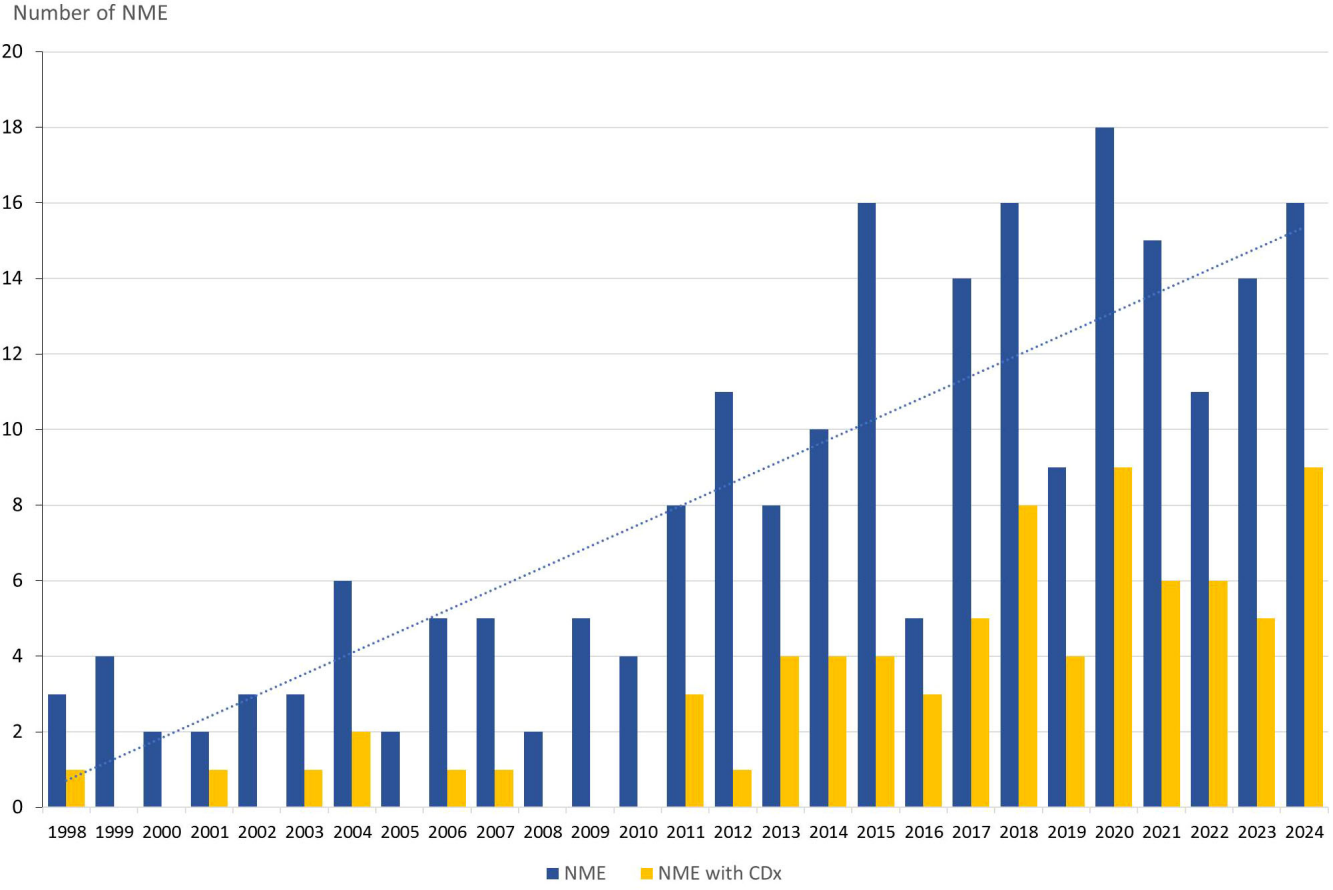
Annual approvals of oncology and hematology NME (blue bar) and the number with a companion diagnostic linked to their use. NME, New Molecular Entities; CDx, Companion Diagnostics.

Between 1998 and the end of 2024, the FDA approved 217 NMEs for the treatment of oncological and hematological malignancies. Forty-six of these approvals were granted from 1998 to 2010, and the remaining 171 NMEs were approved in the subsequent period up to 2025. When the overall therapeutic areas were examined separately, 138 drugs (64%) were approved for oncological indications, and 79 drugs (36%) were approved for hematological indications. Upon classifying NMEs by their molecular/therapeutic class, Kinase Inhibitors emerged as the leading category, comprising 80 NMEs (37%), including drugs like imatinib, crizotinib, and capmatinib. Antibodies formed the second-largest group, with 44 NMEs (20%), encompassing both monoclonal antibodies, like trastuzumab and dostarlimab, and bispecific antibodies, like blinatumomab and amivantamab. Small-molecule drugs ranked third with 31 NMEs (14%), featuring drugs like the PARP inhibitor olaparib and the BCL-2 inhibitor venetoclax. Chemotherapeutics, including cytotoxic agents, like capecitabine and oxaliplatin, accounted for 20 NMEs (9%). Advanced Therapy Medicinal Products (ATMPs) included 12 NMEs (6%), covering cell and gene therapies, like CAR-T cell therapies, including tisagenlecleucel and ciltacabtagene autoleucel. The Antibody-Drug Conjugate (ADC) class comprised 12 NMEs (6%) with drugs like trastuzumab deruxtecan and tisotumab vedotin. Radiopharmaceuticals represented the smallest group with five NMEs (2%), including drugs like iobenguane I-131 and lutetium Lu-177 vipivotide tetraxetan. The “Others” category included various drugs that did not fit into the predefined groups, like immunomodulator pomalidomide and the aromatase inhibitor exemestane, resulting in 13 NMEs (6%). The annual approval rates for oncological and hematological NMEs from 1998 to 2024 showed a consistent upward trend, as illustrated by the linear trend line in **Figure 1**. Comparing the periods 1998–2010 with 2011-2024, the mean annual number of FDA-approved NMEs increased from 3.5 to 12.2 drugs.

### Drugs with companion diagnostics

Among the 217 NMEs approved between 1998 and the end of 2024, 78 (36%) were linked to one or more CDx. For 52 (67%) of the 78 NMEs approved with a CDx assay, both the drug and CDx received approval simultaneously, whereas in the remaining 26 (33%), CDx was approved later through a supplemental process. Kinase Inhibitors are the NME class most frequently paired with a CDx, with 48 (60%) of the 80 drugs, followed by antibodies (39%), and Small Molecule Drugs (26%) (13). As shown in **Figure 1**, particularly after 2010, CDx began to significantly impact the regulatory approval of oncological and hematological NMEs. Comparing the periods 1998–2010 and 2011–2024 reveals a notable increase in NMEs approved with a CDx. During the first period, from 1998 to 2010, seven NMEs were associated with a CDx, accounting for 15% of all newly approved NMEs in oncology and hematology. However, in the subsequent period from 2011 to 2024, this number rose to 71 NMEs, representing 42% of the NME approvals.

### Tissue agnostic drugs

Among the 217 NMEs that have obtained approval by the FDA since 1998, nine (4%) have been approved for a tissue-agnostic indication (10). These approvals are for the treatment of solid tumors that exhibit genetic or proteomic molecular aberrations relevant to a given drug. All the NMEs were associated with a CDx assay for patient selection during clinical development. **Table 1** presents detailed information on these drugs, including their molecular/therapeutic class, indications, and approval dates for both drugs, and their corresponding CDx assays (13). For eight of the nine drugs, approval of the CDx assay was significantly delayed compared to the drug approval date. The mean delay between drug approval and the CDx assay was 707 days, ranging from 0 to 1732 days.

**Table 1:** Details on FDA-approved tissue-agnostic drugs, including molecular/therapeutic classification, indications, and approval dates for both drugs and their corresponding CDx.

Drugs	Molecular/therapeutic class	Tumor agnostic indication	Date drug approval	Date CDx approval	Difference days
Dabrafenib	Kinase inhibitor	*BRAF V600E* mutation solid tumors	06/22/2022	12/31/2024^1^	923
Trametinib	Kinase inhibitor	*BRAF V600E* mutation solid tumors	06/22/2022	12/31/2024^1^	923
Pembrolizumab	Antibody	MSI-H, dMMR, and TMB-H solid tumors	05/23/2017	06/16/2022^2^	1732
Larotrectinib	Kinase inhibitor	*NTRK* gene fusion solid tumors	11/26/2018	10/23/2020	697
Entrectinib	Kinase inhibitor	*NTRK* gene fusion solid tumors	08/15/2019	06/07/2022	1027
Trastuzumab deruxtecan	Antibody-drug conjugate	HER2 positive (IHC3+) solid tumors	04/05/2024	12/31/2024^1^	270
Selpercatinib	Kinase inhibitor	*RET* gene fusion solid tumors	09/21/2022	10/06/2023	380
Dostarlimab	Antibody	dMMR solid tumors	04/22/2021	04/22/2021	0
Repotrectinib	Kinase inhibitor	*NTRK* gene fusion solid tumors	11/15/2023	12/31/2024^1^	412

^1^By 12/31/2024 no CDx assay was approved by the FDA.

^2^The CDx approval date is for the MSI-H assay.

## Interpretation

Since the beginning of the century, the number of NMEs approved by the FDA for the treatment of oncological and hematological malignancies has steadily increased. This trend is largely due to an improved understanding of the pathophysiology and mechanisms of action of the drugs. Much of this progress has been linked to technological advancements in genomic and proteomic analytical and diagnostic methods (1, 3). Understanding tumor biology, especially in terms of heterogeneity and oncogenic drivers, has paved the way for the development of numerous targeted therapies, including kinase inhibitors, mono- and bispecific antibodies, ADCs, and, more recently, ATMPs in the form of various cellular and gene therapy products. Among these novel therapies, kinase inhibitors, which include both single- and multi-target inhibitors, constitute the largest group (37%), followed by antibodies and small-molecule drugs. As the current analysis indicates, this development has notably accelerated over the past 10–15 years, during which the majority of NMEs have received FDA approval.

For a number of NMEs, the CDx assay plays a crucial role in identifying the molecular prerequisites necessary for potential therapeutic effects, thereby ensuring that patients receive appropriate treatment. Among all the NMEs approved up to 2025, 78 (36%) were linked to an FDA-approved CDx assay. However, this proportion varies across the molecular/therapeutic classes. Kinase inhibitors showed the highest percentage (60%), followed by antibodies (39%), while no CDx assays have been approved for patient selection in the categories of chemotherapeutics and ATMPs. Before 2011, only a limited number of NMEs were approved with a CDx, with IHC and *in situ* hybridization (ISH) being the primary analytical methods. The introduction of polymerase chain reaction (PCR) technology in 2011 and next-generation sequencing (NGS) in 2017, as CDx platforms, has significantly transformed the landscape of NMEs associated with CDx assays. Currently, genomic technologies such as PCR and NGS are the dominant analytical platforms for CDx assays (13).

Given the critical importance of CDx, it is crucial that an analytical and clinically validated assay be available and receive regulatory approval alongside the drug to ensure a correct treatment decision (14). Unfortunately, unlike the Japanese regulatory authorities, the FDA has not consistently managed to secure simultaneous approval of drugs and CDx (15, 16). For a particular group of NMSs, specifically tissue-agnostic drugs, it is essential that the CDx assay is accessible simultaneously with the drug, as it is a biomarker that determines the indication (14). In a draft guide document on tissue-agnostic drug development in oncology, the FDA explains that the term refers to a drug that targets a specific molecular alteration across multiple cancer types, as defined, such as organ, tissue, or tumor type (17). Thus, the prescribing of a tissue-agnostic drug is not determined by a traditional histology-based classification but by a taxonomy reliant on tumor molecular aberrations identified through a biomarker/CDx assay. Up to 2025, nine NMEs have been approved for tissue-agnostic drug indications, and the current analysis revealed a mean delay of nearly two years (707 days) between drug approval and the corresponding CDx. A notable example is pembrolizumab, which received FDA approval in May 2017 for the treatment of patients with unresectable or metastatic, microsatellite instability-high (MSI-H), or mismatch repair deficiency (MRRd). However, a CDx assay for this indication was not approved until more than four years later. In February 2022, the FDA approved an NGS assay to detect MSI-H status in patients with solid tumors (13). The most recent FDA approval of a tumor-agnostic drug occurred in April 2024 when trastuzumab deruxtecan was approved for patients with metastatic HER2-positive (IHC 3+) solid tumors (10). Despite the approval of the drug for this indication over a year ago, an assay for this pan-tumor indication remains unavailable (13, 18).

When a targeted drug, including tissue-agnostic drugs, is approved prior to its companion diagnostic (CDx) assay, healthcare providers are often compelled to utilize a local laboratory-developed test (LDT). Prior to using an LDT assay to guide treatment decisions, they must undergo stringent analytical and clinical validation to ensure their quality. However, this requirement has not been met consistently. A publication by the FDA documented several instances of unreliable LDTs that could potentially harm patients (19). Furthermore, subsequent publications have reported various quality issues associated with local LDTs (20–23). Given the pivotal role of CDx assays in the therapeutic decision-making process, it is essential that these assays simultaneously obtain regulatory approval with the associated drug (16). By the end of 2028, all CDx assays used by clinical laboratories within the European Union must have been approved in accordance with the new CE-IVDR. Consequently, local LDTs will no longer be permissible, except in a limited number of special situations (8).

## Summary and conclusion

The number of NMEs used for the treatment of oncological and hematological malignancies has significantly increased over the past 25–30 years. This trend is largely linked to an improved understanding of disease pathophysiology, made possible by advancements in genomic and proteomic analytical technologies. Many of these NMEs are targeted therapies that are specifically designed to interact with molecular targets that play crucial roles in cellular mechanisms, thereby inhibiting the growth and survival of cancer cells. Often, these NMEs are paired with a CDx assay to identify the patient population most likely to respond, and since 2011, this has been true for 42% of all NME approvals. This percentage is anticipated to rise as new molecular analytical technologies, like RNA sequencing and mass spectrometry, are incorporated into the future CDx armamentarium. By definition, a CDx assay is a device or assay that provides essential information for the safe and effective use of a corresponding therapeutic product, which inherently requires its availability alongside the drug it is meant to guide. However, as this analysis revealed, this is not always the situation, particularly for tissue-agnostic drugs. A delay of several years before an analytically and clinically validated CDx assay became available highlights the need for better planning of future drug-diagnostic co-development projects among pharmaceutical companies as well as increased attention from regulators.

## Data Availability

Publicly available datasets were analyzed in this study. This data can be found here: 1. FDA. Oncology (Cancer)/Hematologic Malignancies Approval Notifications. https://www.fda.gov/drugs/resources-information-approved-drugs/oncology-cancerhematologic-malignancies-approval-notifications.2. FDA. Approved Cellular and Gene Therapy Products. https://www.fda.gov/vaccines-blood-biologics/cellular-gene-therapy-products/approved-cellular-and-gene-therapy-products. 3. FDA. List of Cleared or Approved Companion Diagnostic Devices (*In Vitro* and Imaging Tools). https://www.fda.gov/medical-devices/in-vitro-diagnostics/list-cleared-or-approved-companion-diagnostic-devices-in-vitro-and-imaging-tools. 4. FDA. Drugs@FDA: FDA-approved drugs. https://www.accessdata.fda.gov/scripts/cder/daf/.
